# Attenuation of Age-Related Hepatic Steatosis by *Dunaliella salina* Microalgae in Senescence Rats through the Regulation of Redox Status, Inflammatory Indices, and Apoptotic Biomarkers

**DOI:** 10.1155/2020/3797218

**Published:** 2020-05-01

**Authors:** Farouk K. El-Baz, Dalia O. Saleh, Gehad A. Abdel Jaleel, Rehab A. Hussein

**Affiliations:** ^1^Plant Biochemistry Department, National Research Centre (NRC), 33 El Buhouth St. (Former El Tahrir St.), Dokki, Giza, P.O. 12622, Egypt; ^2^Pharmacology Department, National Research Centre (NRC), 33 El Buhouth St. (Former El Tahrir St.), Dokki, Giza, P.O. 12622, Egypt; ^3^Pharmacognosy Department, National Research Centre (NRC), 33 El Buhouth St. (Former El Tahrir St.), Dokki, Giza, P.O. 12622, Egypt

## Abstract

**Background:**

Hepatic steatosis is the most common type of chronic liver disease and is considered an established risk factor of major chronic diseases.

**Purpose:**

The present study aimed to investigate the effect of *Dunaliella salina*, a microalga and its isolated zeaxanthin on age-related hepatic steatosis as well as their underling mechanism. *Study Design*. Age-related hepatic steatosis was induced in rats by intraperitoneal injection of D-galactose (200 mg/kg/day) for eight consecutive weeks. *D. salina* biomass (BDS; 450 mg/kg), its polar fraction (PDS; 30 mg/kg), carotenoid fraction (CDS; 30 mg/kg), and isolated zeaxanthin heneicosylate (ZH; 250 *μ*g/kg) were orally administered to D-galactose treated rats for two weeks.

**Methods:**

Blood samples were collected 24 hours after the last dose of *D. salina* treatments, animals were sacrificed, and liver tissues were isolated. Sera as well as hepatic tissue homogenates were used for further investigations. Liver tissues were also used for histopathological and immunohistochemical examinations. A computed virtual docking study for the biologically active candidates was performed to confirm the proposed mechanism of action.

**Results:**

Oral treatment of D-galactose-injected rats with BDS, PDS, CDS, or ZH ameliorated the serum hepatic function parameters as well as serum levels of adiponectin, apolipoprotein B 100, and insulin. Furthermore, *D. salina* decreased the hepatic lipid contents, redox status biomarkers, inflammatory cytokine, and showing antiapoptotic properties. Molecular docking of *β*-carotene and zeaxanthin on various receptors involved in the pathophysiological cascade of steatosis highlighted the possible mechanism underlying the observed therapeutic effect.

**Conclusion:**

*D. salina* carotenoids have beneficial effect on age-related hepatic steatosis in senescence rats through the regulation of redox status, inflammatory indices, and apoptotic biomarkers.

## 1. Introduction

Nonalcoholic fatty liver disease (NAFLD), a disease that is strictly linked to obesity and insulin resistance (IR), is characterized by hyperinsulinaemia, hypertriglyceridemia, and fatty infiltration of the liver, which is known as hepatic steatosis [1]. Its incidence is more prevalent in older populations, and it varies from simple liver steatosis, through nonalcoholic steatohepatitis (NASH) to advanced fibrosis, cirrhosis, and hepatocellular carcinoma. These three pathologic conditions are accompanied with elevated prevalence and incidence of cardiovascular disease and diabetes mellitus [2]. It has been previously proposed that aging processes may induce hepatic steatosis via various mechanisms, the most important of which are adipose tissue dysfunction, impaired autophagy, and redox status [3].

On the other hand, cellular senescence is a state of permanent cell-cycle arrest correlated with mitochondrial dysfunction and the secretion of proinflammatory cytokines that contributes to age-related tissue degeneration [4]. It has been observed that hepatocytes develop a senescent phenotype during the lifespan of mice [5] and with age-related hepatic disease in humans [6]. However, the association between liver fat accumulation and cellular senescence remains unclear. Herein, it has been hypothesized that cellular senescence due to impaired fat metabolism leads to hepatic steatosis.

Therefore, the goal of the current study was to investigate the effect of *Dunaliella salina*; unicellular marine phytoplankton, that belongs to the phylum Chlorophyta, and one of the richest natural producers of carotenoids particularly *β*-carotene and zeaxanthin, on age-related hepatic steatosis. Likewise, the current investigation aimed to unveil the underlying mechanism by which *D. salina* and major carotenoids exerts their actions. In addition, a docking study was carried out to define the affinity of *β*-carotene and zeaxanthin towards the proposed transcription factor targets confirmed by an in vivo study implemented on hepatic steatosis induced in the aged rat model.

## 2. Materials and Methods

### 2.1. Cultivation and Preparation of Carotenoid Fractions from *D. salina*

#### 2.1.1. Cultivation of *D. salina* in the Photobioreactor

Algal species (*Dunaliella salina*) isolated from salt pond in Al-Fayoum are grown by using Bold nutrient media containing sodium chloride with a concentration of 100g/L for algal isolation and purification [7]. After growing *D. salina* for 10 days under lab conditions, it was then transferred to a vertical photobioreactor with a capacity of 4000 L. Reservoir (1000 L) tank-associated pipe work proprietary in line pigging systems was used for removal of all biofilms. In addition, 10 L basket centrifuge for harvesting was connected to the system. The alga connects the data acquisition system used for online measurements. Tap water was used for the cultivation of algae in the PBR. Water was sterilized using hypochlorite, and after that, sodium thiosulphate was added to remove the excess hypochlorite. The chlorine test was performed to insure no residual chlorine is present. Nutrient solution of Bold was used for growing *D. salina*.

One millilitre per liter of micronutrient solution was added to the culture medium. To insure the purity of the culture, samples are taken regularly and examined microscopically. Carbon dioxide was injected into the culture as a carbon source. The culture is left to grow until the biomass reached the maximum (2–2.5 gm/L). Algal biomass is harvested using the basket centrifuge at 2000 rpm, washed twice with water, and dried in the sun dryer where the temperature reached approximately 45°C and then grounded into homogeneous fine powder.

#### 2.1.2. Preparation of Carotenoid Fractions from *D. salina*

The dried biomass of *D. salina* was extensively milled to ensure the rupture of the cell membrane. The algal biomass was successively extracted. Initially, nonpolar solvent mixture (hexane, ethyl acetate (80 : 20)) was used for extraction by maceration under dim conditions till exhaustion aiming for the carotenoid content. The carotenoid-rich fraction was filtered and dried under reduced pressure in a rotary evaporator apparatus at a temperature not exceeding 40°C till complete dryness and the dried fraction was kept in dark bottles in the refrigerator at a temperature less than 4°C for further analysis. The residue of the microalgal biomass is allowed to dry and further extracted with 70% methanol till exhaustion to render the polar fraction which is filtered and dried under the same conditions as the carotenoid fraction. The carotenoid fraction was subjected to repeated chromatographic analysis for the isolation and purification of zeaxanthin in the form of its heneicosylate ester as previously reported [8].

### 22. Docking Study

#### 2.2.1. Computational Methods

Docking calculations were carried out using DockingServer [9]. The MMFF94 force field was used for energy minimization of ligand molecules (*β*-carotene and zeaxanthin) using DockingServer. Gasteiger partial charges were added to the ligand atoms. Nonpolar hydrogen atoms were merged, and rotatable bonds were defined. Docking calculations were carried out on NF-*κ*B and Nrf2 protein models. Essential hydrogen atoms, Kollman united atom type charges, and solvation parameters were added with the aid of AutoDock tools [10]. Affinity (grid) maps of 20 × 20 × 20 Å grid points and 0.375 Å spacing were generated using the Autogrid program. AutoDock parameter set- and distance-dependent dielectric functions were used in the calculation of the van der Waals and the electrostatic terms, respectively. Docking simulations were performed using the Lamarckian genetic algorithm (LGA) and the Solis and Wets local search method [11]. Initial position, orientation, and torsions of the ligand molecules were set randomly. Each docking experiment was derived from 10 different runs that were set to terminate after a maximum of 250000 energy evaluations. The population size was set to 150. During the search, a translational step of 0.2 Å and quaternion and torsion steps of 5 were applied.

### 2.3. Pharmacological Study

#### 2.3.1. Animals

Male Westar albino rats weighing 130–150 g were obtained from the Animal House Colony of the National Research Centre, housed in plastic cages containing wood shavings, and kept under conventional conditions. The rats were provided with a basal diet and water ad libitum and allowed to acclimatize to the laboratory environment for 7 days before starting the experiment. The experiment was conducted in accordance with ethical procedures was approved by the National Research Centre (Dokki, Giza, Egypt)—Medical Research Ethics Committee for the use of animal subjects.

#### 2.3.2. Chemicals

D-Galactose was purchased from Sigma-Aldrich (St. Louis, Missouri, USA). All other chemicals used were purchased from standard commercial suppliers and were of analytical grade quality.

#### 2.3.3. Experimental Design

Age-related hepatic steatosis was induced in rats by intraperitoneal injection of D-galactose (200 mg/kg/day) for eight consecutive weeks. Thirty albino rats were assigned into five groups; each group contained six rats. Group I received saline and served as a negative control group, and group II received D-galactose for eight weeks and served as a positive control; in addition, groups III, IV, V, and VI received D-galactose for eight consecutive weeks then injected with *D. salina* biomass (BDS; 450 mg/kg; po), its polar fraction (PDS; 30 mg/kg; po), carotenoid fraction (CDS; 30 mg/kg; po), and its isolated zeaxanthin (ZH; 250 *μ*g/kg; po), respectively, for two consecutive weeks. The doses were calculated according to the yields of the fractions and the isolated compound, ZH.

Blood samples were collected 24 hours after the last dose of the *D. salina* treatments, animals were sacrificed, and the liver was isolated. Sera were used to measure the liver functions parameter. Hepatic tissue homogenate was used for the further biochemical analysis. Liver tissues were fixed in 10% formalin for further histopathological and immunohistochemical examinations.

*(1) Biochemical Assessment*. Serum levels of aspartate aminotransferase (AST) and alanine aminotransferase (ALT) were determined according to the methods of Reitman and Frankel [12]. The absorbance was measured at 510 nm, and the results expressed in units per millilitre of serum. Serum insulin, adiponectin, and apolipoprotein B (Apo B) were determined using ELISA, with test reagent kits (EIAab).

Hepatic triglycerides (TG) were measured colorimetrically at 505 nm (492–550 nm) [13]. Similarly, hepatic total cholesterol (TC) was estimated by the enzymatic colorimetric method at 505 nm (492–550 nm) [14].

Catalase (CAT) and glutathione-S-transferase (GST) were assayed according to the methods of Wei and Frenkel [15] and Wilce and Parker [16]. The tissue levels of interleukin-6 (IL-6) were also determined with ELISA using a test reagent kit (Immuno-Biological Laboratories) according to the method of Ferrari et al. [17]. The tissue levels of the cytokine modulator nuclear factor kappa B (NF-*κ*B), nuclear factor like-2 (Nrf2), myeloperoxidase (MPO), and caspase were determined using ELISA, with test reagent kits (EIAab).

#### 2.3.4. Histopathological and Immunohistochemical Assessments

Liver samples were dissected out, excised from the experimental animals of each group, held with the normal saline, fixed in 10% formalin, and processed for paraffin embedding following the microtome technique. The sections were taken at 5 *μ*m thickness, processed in alcohol-xylene series, and were stained with alum-haematoxylin and eosin. The sections were examined microscopically under 40x magnification for the evaluation of histopathological changes.

Sections (5 *μ*m) were prepared and mounted on poly-l-lysine-coated slides. After deparaffinization, antigen retrieval was applied using citrate buffer (0.1 M, pH: 6.0). Hydrogen peroxide (3%) was used for peroxidase activity inhibition. Bcl-2 primary antibodies (Santa Cruz) were used for antigen detection in fixed tissues and probed with a secondary antibody provided by the HRP kit (Labvision). 3-Amino-9-ethylcarbazole (AEC) (Labvision) was used as a chromogen to counterstain the slides, which were evaluated under light microscopy (Nikon Eclipse E-600). For immunopositive cell counting, NIS 4.0 Nikon Image Analysis Software was utilized. The immunopositive cells in four different objective areas were counted under 100x magnification.

#### 2.3.5. Statistical Analysis

Data are presented as mean + SE. Statistical analysis of the data was carried out using one-way analysis of variance (ANOVA) followed by Tukey's multiple comparison test to judge the difference between the various groups. Statistical significance was acceptable to a level of *P* < 0.05. Data analysis was accomplished using the software program GraPad Prism (version 5).

## 3. Results

### 3.1. Docking Study

#### 3.1.1. Docking on Nrf2

**Figure 1:**
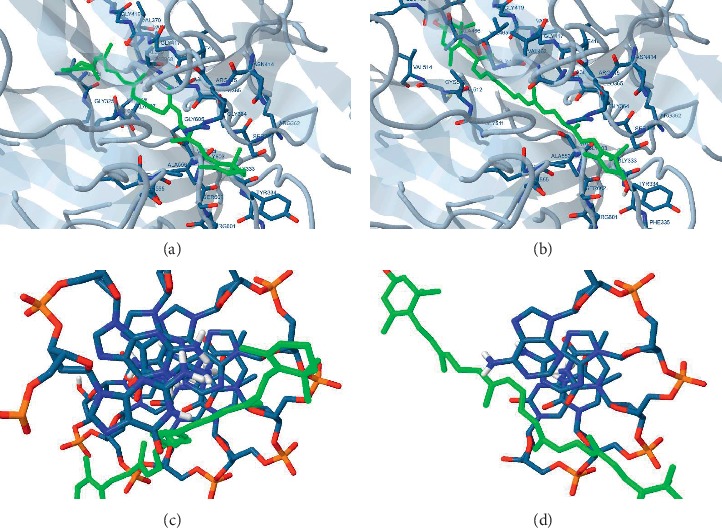
Docking of *β*-carotene and zeaxanthin on Nrf2 (a and b) and NF-*κ*B (c and d).

Both ligands displayed high affinities (−9 kcal/mol and −6.59 kcal/mol for *β*-carotene and zeaxanthin, respectively) with 100% frequency towards the receptor although *β*-carotene showed higher inhibition constant (IC_50_; 252.98 nM and 14.89 nM for *β*-carotene and zeaxanthin, respectively) which reflects higher stability for ligand-receptor complex (Table 1 and Figure 1).

#### 3.1.2. Docking on NF-*κ*B

Zeaxanthin displayed low affinity (0.03 kcal/mol), whereas *β*-carotene displayed moderate affinity (−2.26 kcal/mol with 60% frequency) towards the receptor (Table 1 and Figure 1).

### 3.2. Pharmacological Study

#### 3.2.1. Serum Hepatic Function Parameters

Injection of D-galactose (200 mg/kg; I.P) for eight consecutive weeks led to a marked augmentation in the serum hepatic function parameters, a manifested elevation in the serum levels of ALT and AST by 2.6 and 1.6 folds, respectively. Treatment of D-galactose-injected rats either with BDS (450 mg/kg), PDS (30 mg/kg), CDS (30 mg/kg), or ZH (250 *μ*g/kg) decreased the serum ALT level significantly by 34%, 39%, 49%, and 55% and decreased the serum AST level by 10%, 20%, 26%, and 36%, respectively, as compared with the untreated rats (Table 2).

#### 3.2.2. Hepatic Steatosis Indicators

D-Galactose injection caused a prominent destortion in the hepatic steatosis predictors, evidenced by a reduction in the serum adiponectin level by about 78% as well as an elevation in the serum Apo B100 reaching 3.8 folds. The fasting serum insulin level was also boosted to 5.4 folds. Furthermore, hepatic tissues isolated from D-galactose treated rats showed an augmentation in the hepatic contents of TC and TG by about 4.4 and 5.4 folds, respectively (Table 2).

Oral treatment of age-related hepatic steatosis either with BDS (450 mg/kg), PDS (30 mg/kg), CDS (30 mg/kg) or ZH (250 *μ*g/kg) regulated the serum levels of adiponectin, Apo B100, and insulin, whereas adiponectin levels were elevated by about 2.6, 2.3, 2.4, and 3.1 folds, respectively. Apo B and insulin levels were declined by about 30%, 51%, 49%, and 48%, respectively, and 51%, 67%, 46%, and 66%, respectively, as compared with the untreated group. Moreover, BDS (450 mg/kg), PDS (30 mg/kg), CDS (30 mg/kg), or ZH (250 *μ*g/kg) showed a modulatory effect on the hepatic lipids contents significantly where the hepatic TC and TG was reduced by about 29%, 36%, 51%, and 63% and 18%, 39%, 46%, and 50%, respectively, as compared with D-galactose treated rats (Table 2).

#### 3.2.3. Hepatic Redox Status Biomarkers

Hepatic steatosis was accompanied with an elevation in the hepatic biomarkers of redox status evidenced by a drop in the hepatic catalase and GST levels significantly by about 75% and 57%, respectively, accompanied by an increase in the hepatic MPO level by 3.4 folds. Treatment of hepatic steatosis by BDS (450 mg/kg), PDS (30 mg/kg), CDS (30 mg/kg), or ZH (250 *μ*g/kg) modulated the hepatic level of catalase by 1, 1.3, 1.7, and 2 folds' elevation, respectively; and the hepatic level of GST by about 0.8, 1, 1.2, and 1.3 folds' elevation, respectively. They also showed a decline in the hepatic level of MPO by 29%, 37%, 45%, and 61%, respectively, as compared with D-galactose treated rats (Table 3).

The endogenous antioxidant defense mechanism regulated by Nrf2, a key controller in the redox homeostasis, was intensely abridged by the induction of age-related hepatic steatosis with D-galactose (200 mg/kg I.P) for eight consecutive weeks. Nrf2 showed a decline by about 47% (91.1 ± 3.9 pg/ml vs. 194.9 ± 6.7 pg/ml).

**Figure 2:**
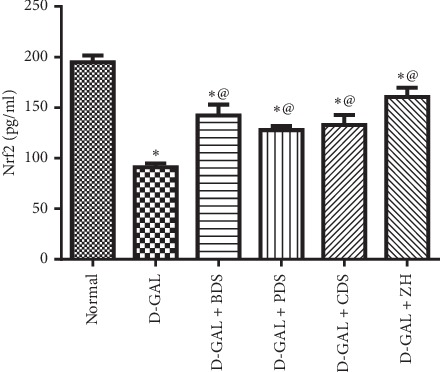
Effect of *D. salina* on hepatic Nrf2 levels in albino rats with age-related hepatic steatosis. Age-related hepatic steatosis was induced in rats by injections of D-galactose (300 mg/kg I.P) 5 days/week for 6 weeks. Rats injected with oral D-galactose for two weeks were treated with BDS (450 mg/kg), PDS (30 mg/kg), CDS (30 mg/kg), or ZH (250 *μ*g/kg). Twenty-four hours later, the last dose of the treatment, rats were sacrificed and liver tissues were homogenized. Tissue homogenate were used for Nrf2 measurement. Data are expressed as mean ± SEM from the normal value. Statistical analysis was performed by one-way analysis of variance (ANOVA) followed by the Tukey–Kramer test for multiple comparisons. ^*∗*^Significantly different from the normal control group at *P* ≥ 0.05. ^@^Significantly different from D-galactose control at *P* ≥ 0.05.

Treatment of hepatic steatosis with BDS (450 mg/kg), PDS (30 mg/kg), CDS (30 mg/kg), or ZH (250 *μ*g/kg) boosted the hepatic Nrf2 levels by about 56%, 40%, 45%, and 76% (142.3 ± 10.7, 127.8 ± 3.8, 132.8 ± 10.1, and 160.4 ± 9.1 pg/ml vs. 91.1 ± 3.9 pg/ml), respectively (Figure 2).

#### 3.2.4. Hepatic Inflammatory Indices

**Figure 3:**
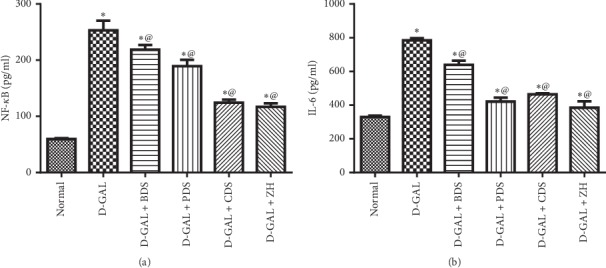
Effect of *D. salina* on hepatic NF-*Κ*B (a) and Il-6 (b) levels in albino rats with age-related hepatic steatosis. Age-related hepatic steatosis was induced in rats by injections of D-galactose (200 mg/kg I.P) for eight weeks. Rats injected with oral D-galactose for two weeks were treated with BDS (450 mg/kg), PDS (30 mg/kg), CDS (30 mg/kg), or ZH (250 *μ*g/kg). Twenty-four hours later, the last dose of the treatment, rats were sacrificed and liver tissues were homogenized. Tissue homogenate was used for IL-6 and NF-*Κ*B measurements. Data are expressed as mean ± SEM from the normal value. Statistical analysis was performed by one-way analysis of variance (ANOVA) followed by Tukey–Kramer test for multiple comparisons. ^*∗*^Significantly different from the normal control group at *P* ≥ 0.05. ^@^Significantly different from D-galactose control at *P* ≥ 0.05.

Hepatic steatosis was similarly allied with an obvious rise in the inflammatory indices evidenced by hepatic NF-*κ*B and IL-6 by about 4.2 folds (253.2 ± 17.1 pg/ml vs. 59.6 ± 1.6 pg/ml) and 2.3 folds (784.6 ± 12.23 pg/ml vs. 329.6 ± 7.2 pg/ml), respectively. Treatment with BDS (450 mg/kg), PDS (30 mg/kg), CDS (30 mg/kg), or ZH (250 *μ*g/kg) reduces the hepatic NF-*Κ*B by about 13%, 25%, 50%, and 54% (218.53 ± 8.6, 189.4 ± 11.2, 124.27 ± 5.5, 117.19 ± 5.9 pg/ml vs. 253.2 ± 17.1 pg/ml), respectively, and a decrease in hepatic IL-6 by about 13%, 21%, and 48%, (639.5 ± 24.2, 421.3 ± 23.5, 464.33 ± 5.9, 384.16 ± 38.5 pg/ml vs. 784.6 ± 12.23 pg/ml), respectively, as compared with the untreated group (Figure 3).

**Figure 4:**
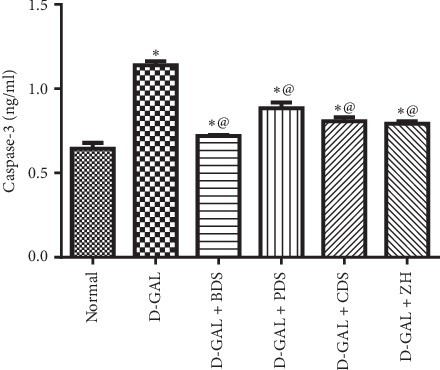
Effect of *D. salina* on hepatic content of caspase-3 in albino rats with age-related hepatic steatosis. Age-related hepatic steatosis was induced in rats by injections of D-galactose (200 mg/kg I.P) for eight weeks. Rats injected with oral D-galactose for two weeks were treated with BDS (450 mg/kg), PDS (30 mg/kg), CDS (30 mg/kg), or ZH (250 *μ*g/kg). Twenty-four hours later, the last dose of the treatment, rats were sacrificed and liver tissues were homogenized. Tissue homogenate were used for caspase assessment. Data are expressed as mean ± SEM from the normal value. Statistical analysis was performed by one-way analysis of variance (ANOVA) followed by the Tukey–Kramer test for multiple comparisons. ^*∗*^Significantly different from the normal control group at *P* ≤ 0.05. ^@^Significantly different from D-galactose control at *P* ≤ 0.05.

Furthermore, steatosis was accompanied with a marked elevation in hepatic caspase-3; a marker of apoptosis by 1.7 folds (1.14 ± 0.02 ng/ml vs. 0.65 ± 0.03 ng/ml). However, BDS (450 mg/kg), PDS (30 mg/kg), CDS (30 mg/kg), and ZH (250 *μ*g/kg) played a modulatory role in caspase-3 indicating antiapoptotic activities to the aforementioned compounds by lowering the caspase-3 by about 37%, 22%, 29%, and 30% (0.72 ± 0.005, 0.89 ± 0.03, 0.81 ± 0.2, 0.79 ± 0.01 ng/ml vs. 1.14 ± 0.02 ng/ml), respectively, as compared with D-galactose-injected rats (Figure 4).

#### 3.2.5. Hepatic Histopathological Examination

Induction of age-related hepatic steatosis with D-galactose (200 mg/kg I.P) for eight weeks showed marked alterations in the histopathological architecture showing dilatation of the central vein and congested inflammatory cellular infiltration at several zones in addition to obliterated sinusoids, necrosis, and cytoplasmic vacuolations of hepatocytes.

Liver sections isolated from rats treated with BDS (450 mg/kg) showed a dilated congested central vein with minimal inflammatory cellular infiltrate with almost preserved hepatic lobules and sections isolated from rats treated with PDS (30 mg/kg) revealing cytoplasmic vacuolations at the level of zone three mainly, dilated congested blood vessels, and noncongested central vein inflammatory cellular infiltrate at the level of portal tracts.

**Figure 5:**
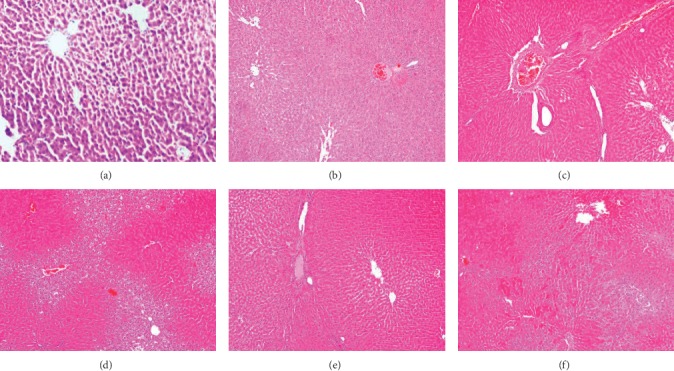
Effect of *D. salina* on hepatic histopathological changes in albino rats with age-related hepatic steatosis. Age-related hepatic steatosis was induced in rats by injections of D-galactose (200 mg/kg I.P) for eight weeks. Rats injected with oral D-galactose for two weeks were treated with BDS (450 mg/kg), PDS (30 mg/kg), CDS (30 mg/kg), or ZH (250 *μ*g/kg). Twenty-four hours later, the last dose of the treatment, rats were sacrificed, and the liver tissue was isolated and fixed with 10% formalin for histopathological examination (40x magnification, H&E).

Similarly, CDS (30 mg/kg) exhibited almost normal hepatic lobules with average sized central vein, while ZH (250 *μ*g/kg) showed thick walled attenuated blood vessels with a preserved hepatic architecture as shown in Figure 5.

#### 3.2.6. Immunohistochemical Hepatic Bcl-2 Assessment

**Figure 6:**
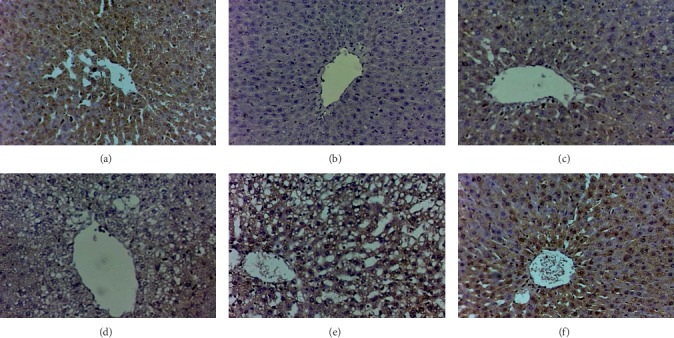
Effect of *D. salina* on hepatic expression of Bcl-2 in albino rats with age-related hepatic steatosis. Age-related hepatic steatosis was induced in rats by injections of D-galactose (200 mg/kg I.P) for eight weeks. Rats injected with oral D-galactose for two weeks were treated with BDS (450 mg/kg), PDS (30 mg/kg), CDS (30 mg/kg), or ZH (250 *μ*g/kg). Twenty-four hours later, the last dose of the treatment, rats were sacrificed and the liver tissue was isolated and fixed with 10% formalin for histopathological examination (100x magnification).

Immunohistochemical Bcl-2 assessment of liver sections of rats with age-related hepatic steatosis showed a significant reduction in the Bcl-2 content; however, the liver sections isolated from rats treated BDS (450 mg/kg), CDS (30 mg/kg), and ZH (250 *μ*g/kg) expressed high degree of positivity revealing improvement. On the other hand, PDS (30 mg/kg) revealed weak scattered stain which signifies much affection with minimal improvement as shown in Figure 6.

## 4. Discussion

**Figure 7:**
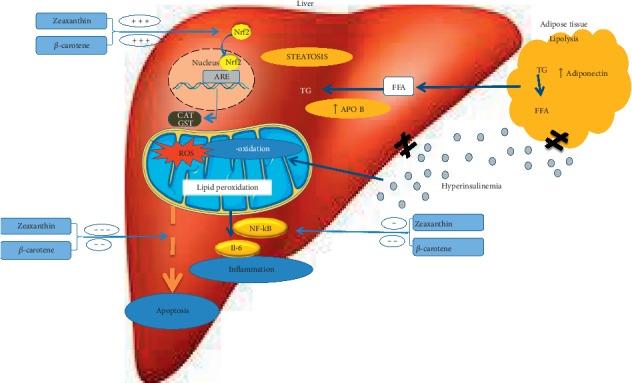
Mechanism of action of *D. salina* microalgae in alleviating age-related hepatic streatosis. ARE : antioxidant response element; CAT: catalase; FFA: free fatty acids; GST: glutathione-S-transferase; IL-6: interleukin-6; NF-*κ*B: nuclear factor kappa beta; Nrf2: NF-E2-related factor 2; ROS: reactive oxygen species; TG: triglycerides.

Hepatic steatosis has been evidenced in the present study with elevated sera liver function biomarkers, ALT and AST levels and increased hepatic TG and TC contents as well as distorted serum levels of insulin, adiponectin, and Apo B100. Adipose tissue plays a pivotal role as an endocrine organ, contributing to energy balance, glucose homeostasis, and inflammation [18]. This relationship has been illustrated in Figure 7. Oral treatment of D-galactose-treated rats with *D. salina* biomass, carotenoid, and polar fractions as well as its isolated zeaxanthin for 2 weeks showed a regulatory role on the hepatic lipid contents significantly where the hepatic TC and TG was reduced, as compared with D-galactose-treated rats. *β*-carotene, a lipid-soluble antioxidant abundant in *D. salina*, has a major role as a precursor of vitamin A, and thus it has a direct influence on cholesterol synthesis [19, 20]. Upon ingestion, the 40 carbon atom carotenoid is cleaved at C15 into retionol, vitamin A, which is further oxidized into retinoic acid. The latter is in turn responsible for the regulation of the expression of genes involved in many metabolic processes [21].

ApoB 100, a dyslipidemia marker and steatosis predictor [22], has been significantly elevated in D-galactose treated rats. Similarly, adiponectin is the most abundant adipose-specific adipokine that reduces liver inflammation and fibrosis. Adiponectin predicts steatosis grade and the severity of hepatic steatosis which is a direct effect or related to the presence of more severe IR and subsequently leads to hyperinsulineamia [23].

Hepatic streatosis is also a major factor of hepatic IR, evidenced in the present study by hyperinsulinaemia. Several clinical trials have correlated the presence of hepatic steatosis with more severe dyslipidemia, hyperinsulinaemia, and IR in the adipose tissue and liver in obese T2 diabetic patients. A recent study has shown that hepatic steatosis-associated IR induces chronic inflammation and IR via an altered protein secretory profile [2]. Oral treatment of rats with age-related hepatic steatosis with *β*-carotenoid and ZH and *D. salina* carotenoid fraction for 2 weeks increased the serum adiponectin levels and reduced the serum Apo B and insulin levels as compared with the untreated group. Similarly, *D. salina* biomass and polar fractions also induced a comparable effect although with modest potencies. It has been previously shown that *β*-carotene and zeaxanthin being potent antioxidants may act as suppressors against the development of IR [24]; thus, it has the ability to reduce the fasting serum insulin levels.

Strong evidences were provided by several studies that the main cause of age-related hepatic steatosis is the accumulation of free radicals in the mitochondria leading to an increase in the redox status and antioxidant defense mechanism [25, 26]. Furthermore, this unbalance provokes various diseases including, chronic liver failure, liver fibrosis, and aging [27]. Oxidative metabolism of D-galactose in the current study contributed to the generation of massive amounts of reactive oxygen species (ROS) in rats [28]. Those byproducts accumulate in cells and lead to osmotic stress as well as elevated redox status, thus causing the acceleration of senescence and aging.

However, the continuous exposure to redox state reduces this ability resulting in occurrence of chronic diseases. Herein, normally, the key controller of redox homeostasis is Nrf2, a transcription factor, which is responsible for the production of endogenous antioxidants that overcome this pathological state [29–31]. Unfortunately, Nrf2 protein is not maintained during aging, at the time of life when the necessity for detoxification is effectively growing, resulting in the occurrence of many chronic diseases [32]. In fact, the previous investigation implemented in our lab has shown a prominent decline in the total hepatic content of Nrf2 in aged rats with a subsequent decrease in the hepatic levels of catalase and GST and elevated level of MPO [33] which is in accordance with this study and others as well [34, 35].

Briefly, the carotenoid fraction of *D. salina* as well as the isolated ZH showed a prominent effect in antagonizing the hepatic steatosis associated with aging. This is attributed to the high content of carotenoids especially zeaxanthin which is also known for its high antioxidant capability. Retinol and carotenoids, especially *β*-carotene possess powerful antioxidant abilities, consequently, prevent the hepatic tissue damage. Considering the potent action of *β*-carotene as a precursor of vitamin A, a potent antioxidant in the combat against ROS [36], probably via its ability to modulate the Nrf2/ARE pathway, is as illustrated in Figure 7.

Likewise, Nrf2 not only is a redox regulator but also plays a major role in linking the cellular response to numerous proinflammatory insults. On the other hand, in rodent models, high-fat diets and obesity have been shown to activate the hepatic inflammatory mediator, NF-*κ*B, which cause hepatic inflammation by an increase in local inflammatory cytokine IL-6. Treatment of age-related hepatic steatosis with *D. salina* microalgae and its isolated zeaxanthin in senescence rats showed a counteracting effect on the hepatic levels of IL-6 and its modulator NF-*κ*B. On the other hand, adiponectin levels that have been elevated in the treated groups are negatively associated with mediators of inflammation, viz, IL-6, which are mainly produced from Kupffer cells and hepatic stellate cells and partly from inflamed hepatocytes. The attenuation of proinflammatory cytokine production by adiponectin is mediated in part by attenuating the translocation of NF-*κ*B to the nucleus [37].

A docking study was also carried out to describe the affinity of *β*-carotene and zeaxanthin towards the crucial regulatory factors. These factors are supposed to be involved in the modulatory effect of *β*-carotene and zeaxanthin on hepatic steatosis, namely, Nrf2 and NF-*κ*B, to assure the proposed mechanism of action of *D. Salina* in attenuating the hepatic steatosis. The high affinity between *β*-carotene found in the carotenoid fraction of *D. salina* and the isolated zeaxanthin with Nrf2 is indicated by the negative energy of binding. This gives a suggestion on the possible direct interaction which is accomplished through the dissociation of the complex of Nrf2/keap releasing the free Nrf2. Thus, it increases the expression of endogenous antioxidants that overcome the imbalanced redox state. On the other hand, the high affinity between *β*-carotene and NF-*κ*B and to a lower extent between zeaxanthin and NF-*κ*B confirms the anti-inflammatory activity of *D. salina* which enables it to reduce the anti-inflammatory cytokine such as IL-6.

Furthermore, excessive apoptosis has been identified during liver aging, in hepatic steatosis and acute and chronic viral hepatitis to combat aging. However, sustained apoptosis has also been linked with the development of hepatic fibrosis. In the present investigation, age-related hepatic steatosis showed an elevation in hepatic caspase-3, a well-known marker of apoptosis. However, *D. salina* exhibited an antiapoptotic activity as compared with untreated senescence rats. Furthermore, Bcl-2 protein expression detected immunohistochemically had confirmed this finding, whereas the liver sections isolated from the treated revealed less apoptotic response.

All of these results have been confirmed by the hepatic histopathological examination as dilatation and congestion were detected in the central as well as the portal veins with inflammatory cells infiltration in the portal zone and degeneration in the hepatocytes all over the parenchyma. These findings were in accordance with [38, 38]. Treatment with *D. salina* carotenoids showed amelioration of the histopathological examination of the liver tissues exhibiting almost normal hepatic lobules with an average sized central vein, while zeaxanthin showed thick-walled attenuated blood vessels with preserved hepatic architecture than appeared sections isolated from rats treated with polar fractions.

## 5. Conclusion

From all the previous results, it can be concluded that *D. salina* microalgae ameliorated age-related hepatic steatosis in senescence rats. These effects are attributed to the inhibitory impact of *D. salina*microalgae and its major constituents, namely, *β*-carotene and zeaxanthin, on the redox status through modulating the Nrf2 pathway, and on the inflammatory indices via the effect on the inflammatory mediator, NF-*κ*B, and eventually on the metabolic homeostasis which in turn alleviate the apoptotic biomarkers.

## Figures and Tables

**Table 1:** Docking of *β*-carotene and zeaxanthin on active sites of different target proteins.

Compounds	Site	Affinity (kcal/mol)	Frequency (%)
*β*-carotene	Nrf2	−9.00	100
	NF-*κ*B	−2.26	60

Zeaxanthin	Nrf2	−6.59	100
	NF-*κ*B	0.03	20

The affinity was expressed as estimated energy of binding in kcal/mol.

**Table 2:** Effect of *D. salina* on hepatic biochemical parameters in albino rats with age-related hepatic steatosis.

Groups	Parameters						
	Serum ALT (U/ml)	Serum AST (U/ml)	Serum insulin (ng/ml/)	Serum adiponectin (*μ*g/ml)	Serum ApoB 100 (*μ*g/ml)	Hepatic TG (mg/g tissue)	Hepatic TC (mg/g tissue)
Normal	47.85 ± 0.62	89.08 ± 4.08	1.125 ± 0.129	7.04 ± 0.728	10.16 ± 0.508	136.68 ± 5.43	36.27 ± 2.70
D-GAL	123.56 ± 10.66^*∗*^	142.3 ± 6.09^*∗*^	6.06 ± 0.61^*∗*^	1.575 ± 0.226^*∗*^	38.59 ± 2.202^*∗*^	747.69 ± 22.35^*∗*^	135.08 ± 13.23^*∗*^
D-GAL + BDS	81.15 ± 4.08^*∗*^^@^	126.7 ± 4.02^*∗*^	2.95 ± 0.22^*∗*^^@^	5.74 ± 0.285^@^	27.07 ± 1.897^*∗*^^@^	611.05 ± 14.58^*∗*^^@^	118.425 ± 9.19^*∗*^^@^
D-GAL + PDS	75.06 ± 4.68^*∗*^^@^	114.02 ± 5.28^*∗*^^@^	2.01 ± 0.17^@^	5.27 ± 1.129^@^	19.03 ± 2.103^*∗*^^@^	458.22 ± 14.62^*∗*^^@^	103.10 ± 9.78^*∗*^^@^
D-GAL + CDS	63.59 ± 6.26^@^	105.87 ± 6.2^@^	3.25 ± 0.33^*∗*^^@^	5.375 ± 0.324^@^	19.73 ± 2.601^*∗*^^@^	401.49 ± 15.99^*∗*^^@^	79.00 ± 6.67^*∗*^^@^
D-GAL + ZH	54.32 ± 5.22^@^	93.10 ± 6.88^@^	2.06 ± 0.02^@^	6.5 ± 0.599^@^	20.25 ± 0.871^*∗*^^@^	374.26 ± 11.14^*∗*^^@^	58.83 ± 5.15^@^

Age-related hepatic steatosis was induced in rats by injections of D-galactose (200 mg/kg I.P.) for eight weeks. Rats injected with oral D-galactose for two weeks were treated with BDS (450 mg/kg), PDS (30 mg/kg), CDS (30 mg/kg), or ZH (250 *μ*g/kg). Twenty-four hours later, the last dose of the treatment, blood samples were collected and sera were used for ALT and AST measurements. Rats were sacrificed and homogenized, and tissue homogenate was used for TG and TC measurements. Data are expressed as mean ± SEM from the normal value. Statistical analysis was performed by one-way analysis of variance (ANOVA) followed by the Tukey–Kramer test for multiple comparisons. ^*∗*^Significantly different from the normal control group at *P* ≤ 0.05. ^@^Significantly different from D-galactose control at *P* ≤ 0.05.

**Table 3:** Effect of *D. salina* on hepatic redox status biomarkers in albino rats with age-related hepatic steatosis.

Groups	Parameters		
	Catalase (U/g tissue)	Hepatic GST (U/g tissue)	Hepatic MPO (U/g tissue)
Normal	0.98 ± 0.08	7.21 ± 0.29	3.15 ± 0.29
D-GAL	0.25 ± 0.016^*∗*^	3.12 ± 0.16^*∗*^	10.71 ± 0.39^*∗*^
D-GAL + BDS	0.48 ± 0.045^*∗*^^@^	5.84 ± 0.07^*∗*^^@^	7.60 ± 0.70^*∗*^^@^
D-GAL + PDS	0.56 ± 0.034^*∗*^^@^	6.22 ± 0.10^*∗*^^@^	6.71 ± 0.16^*∗*^^@^
D-GAL + CDS	0.65 ± 0.02^*∗*^^@^	6.72 ± 0.22^@^	5.89 ± 0.57^@^
D-GAL + ZH	0.78 ± 0.049^*∗*^^@^	7.24 ± 0.31^@^	4.18 ± 0.31^*∗*^^@^

Age-related hepatic steatosis was induced in rats by injections of D-galactose (200 mg/kg I.P) for eight weeks. Rats injected with oral D-galactose for two weeks were treated with BDS (450 mg/kg), PDS (30 mg/kg), CDS (30 mg/kg), or ZH (250 *μ*g/kg). Twenty-four hours later, the last dose of the treatment, rats were sacrificed, liver tissue was isolated and homogenized, and the tissue homogenate was used for hepatic catalase, GST, and MPO measurements. Data are expressed as mean ± SEM from the normal value. Statistical analysis was performed by one-way analysis of variance (ANOVA) followed by the Tukey–Kramer test for multiple comparisons. ^*∗*^Significantly different from the normal control group at *P* ≤ 0.05. ^@^Significantly different from D-galactose control at *P* ≤ 0.05.

## Data Availability

All the data used to support this study will be made available from the corresponding author upon request.

## Abbreviations:

ALT: Alanine aminotransferase
ANOVA: One-way analysis of variance
ARE: Antioxidant response element
AST: Aspartate aminotransferase
BDS: *Dunaliella salina* biomass
CAT: Catalase
CDS: *Dunaliella salina* carotenoid fraction
GST: Glutathione-S-transferase
IL-6: Interleukin-6
IR: Insulin resistance
MPO: Myeloperoxidase
NAFLD: Nonalcoholic fatty liver disease
NASH: Nonalcoholic steatohepatitis
NF-*κ*B: Nuclear factor kappa B
Nrf2: Nuclear factor like-2
PDS: *Dunaliella salina* polar fraction
TC: Total cholesterol
ROS: Reactive oxygen species
TG: Triglycerides
ZH: Zeaxanthin.
